# A Genome-Wide Association Study Coupled With a Transcriptomic Analysis Reveals the Genetic Loci and Candidate Genes Governing the Flowering Time in Alfalfa (*Medicago sativa* L.)

**DOI:** 10.3389/fpls.2022.913947

**Published:** 2022-07-11

**Authors:** Fei He, Fan Zhang, Xueqian Jiang, Ruicai Long, Zhen Wang, Yishi Chen, Mingna Li, Ting Gao, Tianhui Yang, Chuan Wang, Junmei Kang, Lin Chen, Qingchuan Yang

**Affiliations:** ^1^Institute of Animal Science, Chinese Academy of Agricultural Sciences, Beijing, China; ^2^Center for Monitoring of Agricultural Ecological Environment and Quality Inspection of Agricultural Products of Tianjin, Tianjin, China; ^3^Institute of Animal Science, Ningxia Academy of Agricultural and Forestry Sciences, Yinchuan, China

**Keywords:** alfalfa, GWAS, flowering time, SNP, haplotypes

## Abstract

The transition to flowering at the right time is very important for adapting to local conditions and maximizing alfalfa yield. However, the understanding of the genetic basis of the alfalfa flowering time remains limited. There are few reliable genes or markers for selection, which hinders progress in genetic research and molecular breeding of this trait in alfalfa. We sequenced 220 alfalfa cultivars and conducted a genome-wide association study (GWAS) involving 875,023 single-nucleotide polymorphisms (SNPs). The phenotypic analysis showed that the breeding status and geographical origin strongly influenced the alfalfa flowering time. Our GWAS revealed 63 loci significantly related to the flowering time. Ninety-five candidate genes were detected at these SNP loci within 40 kb (20 kb up- and downstream). Thirty-six percent of the candidate genes are involved in development and pollen tube growth, indicating that these genes are key genetic mechanisms of alfalfa growth and development. The transcriptomic analysis showed that 1,924, 2,405, and 3,779 differentially expressed genes (DEGs) were upregulated across the three growth stages, while 1,651, 2,613, and 4,730 DEGs were downregulated across the stages. Combining the results of our GWAS and transcriptome analysis, in total, 38 candidate genes (7 differentially expressed during the bud stage, 13 differentially expressed during the initial flowering stage, and 18 differentially expressed during the full flowering stage) were identified. Two SNPs located in the upstream region of the *Msa0888690* gene (which is involved in isop renoids) were significantly related to flowering. The two significant SNPs within the upstream region of *Msa0888690* existed as four different haplotypes in this panel. The genes identified in this study represent a series of candidate targets for further research investigating the alfalfa flowering time and could be used for alfalfa molecular breeding.

## Introduction

Alfalfa (*Medicago sativa* L.) is a global forage legume crop species with a high yield and nutritional value (He et al., 2019). According to the growth and development stages of alfalfa, it is mainly divided into the following stages: greening stage, branching stage, budding stage, flowering stage and maturity stage. The flowering stage period is an important trait that can determine when alfalfa is harvested, and different varieties show extensive variation in life-history characteristics, such as the flowering time. The flowering time is a quantitative trait that is determined by genetic and environmental factors (Koornneef et al., 1998a). In plants, early flowering may be a desirable trait in practical production because it can hasten the growing season, thus avoiding unfavorable climatic conditions (such as drought) (Ridge et al., 2017). The flowering time is also very important for alfalfa because it can determine the harvest time, which affects the forage quality, silage, and yield of alfalfa (Adhikari et al., 2019). Alfalfa differs from other grass species; alfalfa is cut several times a year, and its total digestible nutrients (TDNs) gradually decrease with increasing maturity. Therefore, alfalfa is usually harvested before the seeds are fully mature. Due to these properties, farmers can choose the most suitable grazing periods and harvest dates, thus maximizing economic benefits (Arzani et al., 2004). Although the flowering time is very important, the potential molecular mechanism controlling the flowering time of alfalfa has not been clarified.

The genetic and genomic basis of the flowering time has been investigated extensively in several plant species, such as *Arabidopsis thaliana*, rice (*Oryza sativa* L.), and wheat (*Triticum aestivum* L.) (Grillo et al., 2013; Hori et al., 2016; Ivanièová et al., 2016), whereas such information in alfalfa is scarce. Many flowering time-related genes, such as Flowering Locus T (*FT*), Flowering Locus C (*FLC*), Constans (*CO*), and suppressor of overexpression of constans 1 (*SOC*1), have been cloned. These genes are mainly responsive to pathways involving the photoperiod, temperature and gibberellin (Lyons et al., 2015; Kang et al., 2019). The *FT* gene is generally considered to integrate inputs of several pathways that ultimately result in the floral transition. In *A. thaliana, FT* loss-of-function mutations result in late flowering under long-day conditions (Koornneef et al., 1998b). The genetic basis of the variation in the flowering time of legume species, such as pea (*Pisum sativum*), soybean (*Glycine max* L.) and *Medicago truncatula*, has been studied. In *M. truncatula*, a significant number of genes have been verified to control the flowering time, including *FTa*, *FTb*, and *FTc* (Hecht et al., 2005). Florigen members of the *FT* family (*MtFTa*1, *MtFTb*1, and *MtFTc*) that control the flowering traits of *M. truncatula* were shown to successfully rescue late-flowering mutant plants by inducing early flowering (Laurie et al., 2011). Similarly, the expression of several genes in *M. sativa*, including *MsLFY* (Zhang et al., 2013), *SPL*13 (Gao et al., 2018), and *MsZFN* (Chao et al., 2014), has been measured; these genes have been molecularly cloned, and their involvement in the flowering time of alfalfa was shown via reverse genetic methods.

Knowledge of single-nucleotide polymorphism (SNP) markers of related traits has become the genetic basis of various trait improvements in plant breeding (Battenfield et al., 2018). In recent years, with the introduction of high-density marker arrays, genome-wide association studies (GWASs) have been actively applied to many different crop species and varieties; SNPs related to many agronomic traits, such as the flowering time (Navarro et al., 2017), salt tolerance (Patil et al., 2016), and plant height (Zhang J. P. et al., 2015), have been found, and many candidate genes or genomic regions have been revealed. For example, eight candidate genes related to the flowering time, including *Hd1*, have been identified in 950 different rice varieties (Huang et al., 2012). Ten candidate genes, including *SOC1*, were identified through an association analysis of 309 soybean varieties (Zhang J. P. et al., 2015). Kim et al. conducted a GWAS of 2,662 soybean varieties and found 18 candidate genes involved in 6 main flowering pathways (Kim et al., 2020). Through a GWAS analysis of 137 core germplasms used worldwide, Shen et al. (2020) found genes related to the flowering time in alfalfa; these genes promoted the transition from vegetative growth to flowering (Shen et al., 2020). These findings provide valuable information for various breeding plans focusing on the flowering time. In addition, RNA sequencing (RNA-seq) is a powerful tool used to discover candidate genes and target traits and has been widely applied to many plant species.

Although many GWASs have been performed in alfalfa, most focused only on drought resistance (Zhang T. J. et al., 2015), salt tolerance (Yu et al., 2016), and forage quality (Lin et al., 2020), while few focused on genetic analyses and the molecular regulatory mechanisms underlying flowering time traits in alfalfa. In addition, due to the lack of a reference genome for alfalfa, most previous studies used the genome of *M. truncatula* as a reference (Wang et al., 2020). However, because these two legumes are different species, few functional genes are found in cultivated alfalfa, limiting improvement in cultivars (Chen et al., 2021). With the development of genome assembly technology, the genomes of the Zhongmu No.1, XinJiangDaYe and Zhongmu-4 varieties have been released (Chen H. et al., 2020; Shen et al., 2020; Long et al., 2022). These findings will greatly facilitate the identification of functional genes involved in the alfalfa flowering time and provide important information for the improvement of alfalfa in the future.

To better understand the genetic structure of the alfalfa flowering time, in this study, we first conducted a GWAS of a panel that consisted of 220 alfalfa varieties. Then, RNA-seq analysis was used to identify the genes involved in the flowering time in alfalfa. Finally, we identified candidate genes associated with the flowering time through a combination of GWAS and RNA-seq analysis. Candidate genes or homologous *A. thaliana* genes with known functions were also proposed. This study enriches our knowledge of the genetic basis underlying the flowering time in alfalfa and provides valuable markers for the molecular breeding of alfalfa.

## Materials and Methods

### Plant Materials and Growth Conditions

The plant materials used in this study included 220 accessions collected worldwide. The sources of the materials were reported in a previous study (Chen et al., 2021). In October 2017, seeds of 220 accessions were planted in a greenhouse of the Chinese Academy of Agricultural Sciences (CAAS) in Langfang, Hebei Province, China (39.59°N, 116.59°E). The greenhouse was maintained such that the photoperiod was 16 h days/8 h nights, the temperature was 22°C, and the relative humidity was 40%. In the experimental field used, the annual average temperature was 11.9°C, the coldest month was January (−4.7°C), and the warmest month was July (26.2°C). The average annual precipitation was 554.9 mm, with large spatial and seasonal variations. During the summer season (July through September), the area received more than 50% of the annual precipitation. The soil was a medium loam that comprised 1.69% organic matter (pH = 7.37). Individual plants were transplanted into the field of the CAAS in April 2018. The field trial employed a randomized complete block design with three replications. Each replication included five cloned plants per individual, and each replicate was spaced 30 cm apart. The spacing was 65 cm between rows and 65 cm between plants. No fertilizer or irrigation was applied, and weeding was performed manually. The remaining 5 cm of mowing was performed in each individual plant before the winter, thus ensuring consistency between individuals.

### Phenotyping of the Flowering Time

The flowering time (the date when the first flower appeared) in the first cycle (daily from April to May) in Langfang in 2019, 2020, and 2021 was measured and converted to photothermal units (PTUs) based on the methods described by Grabowski et al. (2017). The average daily temperature (avgT) was calculated as avgT = (minT + maxT)/2. MinT represents the lowest temperature, and maxT represents the highest temperature. In the PTU calculation formula, days were defined as the total days from the date accumulating after 5 consecutive days during which avgT was >10°C to the date the first flower appeared. dayL was defined as the total number of hours between sunrise and sunset in a day. The information of avgT and dayL was obtained from the weather station of Yi Kang Nong (Beijing, China). The random-effects model used was described by Zhang et al. (2020). The frequency distribution, coefficient of variation (CV), broad-sense heritability (*H*^2^), and analysis of variance (ANOVA) of the flowering time traits among the accessions were estimated using SPSS v17.0 according to the methods described by Saxena et al. (2014). The three-year average phenotypic value was recorded as FT, and the data from each individual year were recorded as FT2019, FT2020, and FT2021.

### Sequencing and Single-Nucleotide Polymorphisms Calling

Phenotyping was performed in 15 individuals per accession, and one individual exhibiting a typical phenotype was selected for sequencing. Young leaves of plants in the early regrowth stage were collected. A CWBIO Plant Genomic DNA Kit (CoWin Biosciences, Beijing, China) was used to extract DNA from 100 mg of fresh young leaf tissue. Paired-end sequencing (with an insert size of approximately 300 bp) was subsequently performed on an Illumina NovaSeq 6000 instrument by Berry Genomics. For the basic information of the sequencing coverage, read count, mapping read, etc., please refer to Long et al. (2022). The raw sequencing data were first subjected to quality filtering via Trimmomatic software with the default parameters (Bolger et al., 2014). BWA-MEM was used to map paired-end sequencing reads to a haploid alfalfa reference genome with 8 chromosomes (Long et al., 2022). SAMtools software was then used to compare and sort the sequencing data, convert the data to a BAM file, and remove duplicates, and the default parameters were used (Li et al., 2009). For information concerning the SNP detection and filtering and the distribution of the generated SNPs on chromosomes, refer to Chen et al. (2021).

### Population Structure, Linkage Disequilibrium and Genome-Wide Association Study

The population structure is an important factor affecting GWAS of complex traits. In this study, ADMIXTURE was used to determine the optimal number of subpopulations (K) for investigating the population genetic structure of the global alfalfa panel, and the LD information was calculated using the software PopLDdecay with the default parameters. The results showed that K = 4 had the lowest value of cross-validation error, suggesting that the 220 alfalfa varieties could be divided into four subgroups (Chen et al., 2021). The GWAS analysis of the flowering time (FT) was performed using BLINK C software using Bayesian-information and linkage-disequilibrium iteratively nested keyway (BLINK) (Huang et al., 2019). This method can improve the statistical power, reduce the computing time and simultaneously reduce the number of false positives and false negatives. Compared with PLINK or FarmCPU, association analyses of BLINK can reveal more genetic loci and more true positives, including loci previously verified in other studies (Huang et al., 2019). In total, 875,023 high-quality SNPs based on the Zhongmu-4 reference haploid genome were used in this study. The threshold for significantly associated loci was a logarithm of odds (LOD) score ≥5.

### RNA-Seq and Transcriptomic Analysis

The total RNA was extracted by using TRIzol reagent (Invitrogen, CA, United States). All RNA extracts were treated with an RNase-Free DNase Set (Qiagen, Valencia, CA, United States) and then cleaned with a RNeasy Mini Kit (Qiagen). The cDNA library construction and sequencing on an Illumina HiSeq 2000 platform were conducted by Novogene (Beijing, China). The flower buds of the early flowering alfalfa variety Cangzhou (the earliest flowering variety among the 220 materials) and the alfalfa representative variety Zhongmu No.1 were sampled at three development stages (the bud stage, initial-flowering stage and full-flowering stage). The flowering time during the three stages occurred at approximately the end of April, the beginning of May and middle and late May. There were two replications per material. The Illumina sequencing of the pooled RNA-seq libraries yielded 24 FASTQ files of sequence data. Clean reads obtained via standard procedures were mapped to the Zhongmu-4 reference haploid genomes by HISAT2 software. Feature counts were used to count the mapped reads before proceeding to the differential expression analysis. The differential expression analysis was performed using DESeq2 software. The gene expression levels were calculated and normalized by reads per kilobase of exon model per million mapped reads (RPKM) (Mortazavi et al., 2008). RNA-seq data were grouped according to different flowering time, and the two groups were compared. The expression values were normalized by scaling to the default setting of 10 million reads. Moderated t statistics for pairwise contrasts were calculated using the Baggerly’s test (Baggerly et al., 2003). The Baggerly’s test (beta-binomial) is a weighted t-type test statistic that compares the proportions of counts in a group of samples against those of another group, giving them different weights depending on their sizes (total counts) (Baggerly et al., 2003). Normalized RPKM values were analyzed by Baggerly’s test to look for significant differences in expression data of Cangzhou alfalfa and Zhongmu No.1 alfalfa samples. Genes with no counts in all two replicates for at least one of the flowering times were discarded as not detectable above the background. Therefore, baggerly’s test is selected for data analysis. The Baggerly’s *p* values were corrected for multiple testing for each contrast separately by means of false discovery rate (FDR) (Benjamini and Hochberg, 1995) for significant genes based on ANOVA. FDR corrected *p*-value <0.05 and log2 of fold change ≥1 was used as a cutoff. Significant differentially expressed genes (DEGs) were identified according to FDR. The functional annotations were performed using the web version of the evolutionary genealogy of genes: Nonsupervised Orthologous Groups (eggNOG) tool.

### Candidate Gene Analysis

Reported alfalfa Zhongmu-4 reference haploid genomes were used to identify genes with significantly associated loci. EggNOG^1^ databases were used for the gene annotations. All genes within 40 kb (20 kb up- and downstream) of the significant loci were identified according to the linkage disequilibrium (LD) of the association panel. We compared the sequences of the identified genes with the *A. thaliana* genome sequence to identify their orthologues based on sequence similarity and ultimately identified 95 genes related to growth and development. The genes near significantly associated loci received increased attention. We identified a total of 1585 differentially expressed genes from three periods of RNA-seq data. Combining the results of our GWAS and transcriptome analysis, in total, 38 candidate genes (7 differentially expressed during the bud stage, 13 differentially expressed during the initial flowering stage, and 18 differentially expressed during the full flowering stage) were identified. Among the 38 candidate genes, three genes were all differentially expressed at the budding stage, the first flowering stage and the full flowering stage. We used these three genes for subsequent verification.

### Pattern Analyses of Candidate Genes by Real-Time Quantitative RT–PCR

The total RNA was extracted from the flowers of normal-growing Zhongmu No.1 and Cangzhou alfalfa with TRIzol reagent according to the manufacturer’s instructions. Then, a cDNA library was constructed for the subsequent reactions. qRT-PCR primers were designed via the NCBI website^2^. The alfalfa actin gene was used for data normalization, and for each sample, three technical replicates were included. The data were quantified by the 2^–(ΔΔ*CT*)^ method (Livak and Schmittgen, 2001).

## Results

### Phenotypic Data Analysis

In 2019, 2020, and 2021, the average PTU values were 204.21, 168.29, and 174.98, respectively, while the CVs during the same years were 11.30, 13.70, and 13.10%, respectively (Table 1). *H*^2^ was calculated as described in a previous study (Tornqvist et al., 2018) and ranged from 0.51 to 0.59. The *P* values of the Kolmogorov–Smirnov normality test were lower than the threshold (0.05) within three years, and the results show that the assumption of the non-normal distribution of PTUs in this associated group was correct. The three-year total PTU and single-year PTU are shown in Supplementary Figures 1, 2. In the subsequent GWAS analysis, the average PTU value across the three years was used. The data showed that the characteristics were normally distributed (Supplementary Figures 1, 2). The flower time correlations between accession means are shown in Supplementary Figure 3. In 2019, 2020, and 2021, the correlation values ranged between 0.15 and 0.38 (Supplementary Figure 3).

**TABLE 1:** Summary statistics of the PTUs of the flowering time in the GWAS population in 2019, 2020, and 2021.

Year	n	Mean	Range	CV (%)	Kurtosis	Skewness	*P* value	*H* ^2^
2019	220	204.21	131.81-271.01	11.30	0.404	0.107	<0.01	0.55
2020	220	168.29	126-254	13.70	0.308	0.662	<0.01	0.59
2021	220	174.98	98.98-291.40	13.10	−0.15	0.597	<0.01	0.51

**FIGURE 1:**
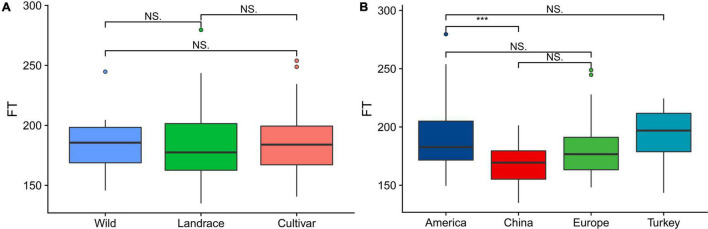
Boxplots showing the variation in the flowering time evaluated in each association panel. **(A)** Boxplot of the flowering time of plants in the subgroups (wild, landrace, and cultivar groups) according to the breeding status. **(B)** Boxplot of the flowering time of plants in the subgroups (America, China, Europe, and Turkey) according to geographical origin. The different asterisks above the boxplots in **(A,B)** indicate significant differences at the P < 0.05 level according to Duncan’s multiple comparison test.

From a breeding perspective, our association group could be divided into the following three subgroups: wild, landrace and cultivar subgroups. The statistical analysis of the flowering time showed that there was no significant difference among the three subgroups. Among the plants in the three subgroups, those in the landrace group had the earliest flowering time, followed by those in the cultivar and wild groups (Figure 1A). The association panel could also be divided into four subgroups according to geographical origin as follows: America, China, Europe, and Turkey. Regarding the flowering time, the American varieties and Chinese varieties exhibited significant differences (*P* < 0.01), while the other three subgroups did not significantly differ, and the flowering time of the Chinese varieties occurred the earliest, followed by the European and American varieties. The Turkish varieties had the latest flowering time (Figure 1B).

### Genome-Wide Association Studies and Candidate Genes for Flowering Time

**FIGURE 2:**
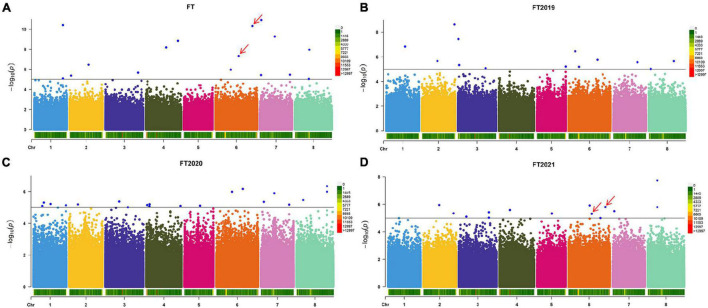
Manhattan plot of the flowering time in different years. **(A)** Manhattan plot of the average flowering time over three years. **(B)** Manhattan plot of the flowering time in 2019. **(C)** Manhattan plot of the flowering time in 2020. **(D)** Manhattan plot of the flowering time in 2021. The GWAS was performed by BLINK C software, and the threshold for significantly associated loci was an LOD score ≥5 (blue line). The red arrow indicates the SNP sites co-located with two years of phenotypic data.

Based on the resequencing results, we obtained 875,023 high-quality SNPs distributed across 8 alfalfa chromosomes. Using three years of phenotypic data to analyze the flowering time, we found a total of 16 significant loci according to the LOD >5.00 threshold (Figure 2A and Supplementary Table 1). These 16 markers are distributed on all chromosomes, except for chromosome 5. Regarding the flowering time, the proportion of phenotypic variance explained (PVE) by the 16 significant SNPs ranged from 3.52 to 9.58%. To determine the relationship between the average of the three years and a single year, we performed a GWAS of the other three phenotypes (FT2019, FT2020, and FT2021) to obtain more association data. We detected 13 markers showing a significant association with the FT2019 phenotype, among which chr2__80931183 was the most significant (−log *p* > 8.63), explaining 7.60% of the variation (Figure 2B and Supplementary Table 1). We detected 20 markers showing a significant association with the FT2020 phenotype (Figure 2C and Supplementary Table 1). We detected 14 markers showing a significant association with the FT2021 phenotype, among which chr8__23871223 was the most significant (−log *p* > 7.74) and explained 15.63% of the variation, and the proportion of PVE ranged from 7.58 to 15.63% (Figure 2D and Supplementary Table 1). A comparison of the markers detected in the different phenotypes showed that the two markers, namely, chr6__58766748 and chr6__92897252, were associated with a two-year phenotype. These two markers were detected in FT and FT2021 and explained 8% of the phenotypic variation, indicating that these markers have a major effect on the alfalfa flowering time. The SNP effect size ranged between 19.25 and 31.42.

**FIGURE 3:**
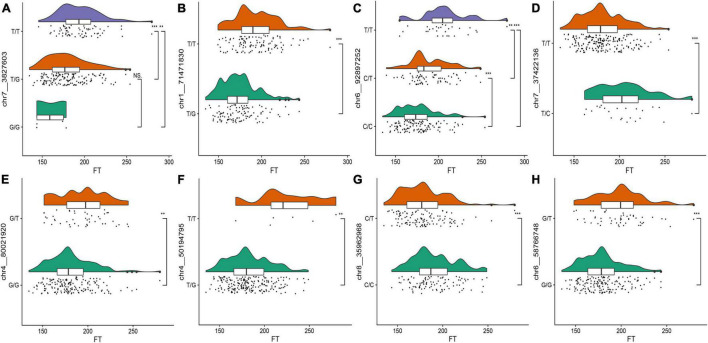
**(A–H)** Raincloud plots of the highest distribution of the flowering time of the plants with relevant SNP genotypes. The top plots represent the kernel density estimation, the middle plots represent box diagrams, and the bottom plots are dithering scatter diagrams. The different asterisks on the right side of the diagram indicate significant differences at the *P* < 0.05 level according to Duncan’s multiple comparison test. The abscissa represents flowering time and the ordinate represents SNP genotype.

To observe the effects of different alleles on the flowering time, we selected the eight SNPs with the highest threshold in FT for observations. Raincloud plots of the genotypes of the eight SNPs associated with the flowering time were constructed (Figure 3). The differences in the flowering time among the different genotypes can be observed in the eight raincloud plots of the SNPs. There is no homozygous genotype *G/G* for SNPs chr1__71471830 or chr4__50194795, and there is no homozygous genotype *C/C* for SNP chr7__37422136. In addition, there is no homozygous genotype *T/T* for chr8__35962968, chr4__80021920 or chr6__58766748. Among the 8 SNPs, 37.5% (3/8) of the *G/G* genotype was related to early flowering, 50% (4/8) of the genotypes were *T/T*, and flowering occurred relatively late. Therefore, we speculate that these two genotypes may be related to the flowering time of alfalfa. To avoid missing any associations, we selected all 63 significant markers detected in the various phenotypes for further characterization. We used the Zhongmu-4 reference genome and identified all genes within 40 kb (20 kb upstream and downstream) of significant loci according to the LD of the association panel. We compared the sequences of the identified genes with the *A. thaliana* genome sequence to identify their orthologues based on sequence similarity and ultimately identified 95 genes related to growth and development for the subsequent analysis (Supplementary Table 2).

**FIGURE 4:**
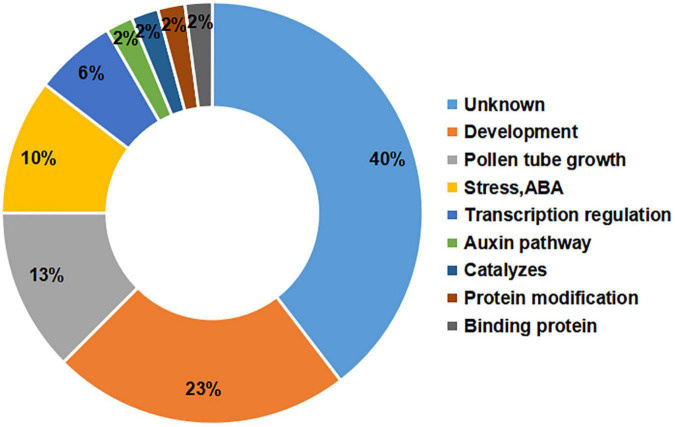
Functional annotations of candidate genes. The number on the left represents the percentage of candidate genes. The text on the right represents the functional classification of the genes.

The gene annotations showed that the greatest percentage of the candidate genes (40%) had unknown functions, but there were large percentages of genes involved in development (23%) or pollen tube growth (13%) (Figure 4). Among the candidate genes, it has been reported that the orthologues of *Msa0041180* and *Msa0041190* regulate plant development (Bekh-Ochir et al., 2013; Ramón et al., 2020). The other genes are involved in pollen tube development and influence flowering. For example, *Msa1016440* (*AT5G41990*) and *Msa1027410* (*AT4G35420*) have been shown to influence flowering during plant development (Tang et al., 2009; Tsuchiya and Eulgem, 2010). In addition, several genes were involved in the auxin pathway, protein modification, and binding proteins, each with a percentage of 2%. The relatively large percentage of genes involved in development and pollen tube growth indicates that our experiments successfully identified genes critical for the alfalfa flowering time.

### RNA-Seq With Respect to the Flowering Time

**FIGURE 5:**
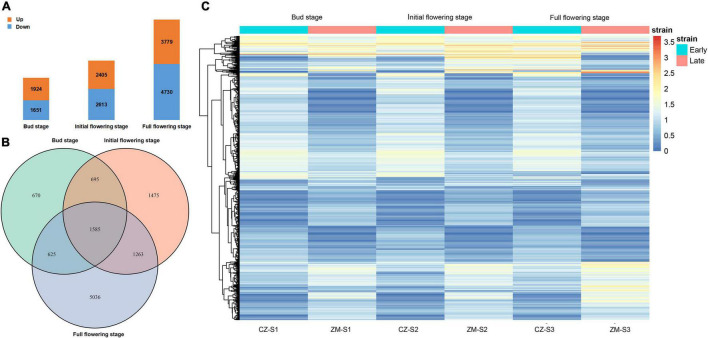
Transcriptomic analysis. **(A)** Number of upregulated genes (orange) and downregulated genes (blue) during alfalfa flowering at three different periods. **(B)** Upregulated and downregulated DEGs in the three periods. **(C)** Heatmap clustering of all DEGs according to their expression level. The different colors indicate different levels of expression. The blue box above represents the early flowering varieties, and red represents the late-flowering varieties.

We collected flower buds from two samples at three growth and development stages, constructed a cDNA library and sequenced the transcriptome. The resulting datasets were named CZ_S1 (Cangzhou alfalfa at the bud stage); CZ_S2 (Cangzhou alfalfa at the initial-flowering stage); CZ_S3 (Cangzhou alfalfa at the full-flowering stage); ZM_S1 (Zhongmu No.1 at the bud stage); ZM_S2 (Zhongmu No.1 at the initial-flowering stage); and ZM_S3 (Zhongmu No.1 at the full-flowering stage). In total, 1,924, 2,405, and 3,779 DEGs were identified as upregulated at the bud, initial-flowering, and full-flowering stages, respectively, and 1651, 2613 and 4730 DEGs were downregulated, respectively (Figure 5A and Supplementary Tables 3–8). Of these DEGs during the three periods, 1585 were common during the flowering period (Figure 5B). An overview of the expression of these DEGs is shown in a heatmap, and their expression could be approximately divided into five patterns (Figure 5C). The Gene Ontology (GO) analysis showed that these DEGs (Supplementary Figure 4) and three periods (Supplementary Figures 5–7) could be divided into the following three GO categories: biological processes, cellular components and molecular functions. In the biological process category, the two largest subcategories during the bud stage were cellular process and single-organism process, and during the initial flowering and full-flowering stages, the DEGs were mostly associated with cellular processes and metabolic processes. In the cellular components category, the two largest subcategories were cell and cell part during all stages. And the two largest subcategories of the molecular function category were catalytic activity and binding.

### Candidate Gene Analysis of the Flowering Time in Alfalfa

**FIGURE 6:**
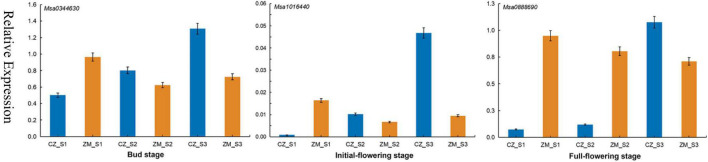
qRT–PCR analysis of three candidate genes in three stages associated with the flowering time in alfalfa.

We combined the results of the Genome-Wide Association Studies and Differentially Expressed Genes analysis to further analyze the genes associated with the flowering time in alfalfa. According to the LD, in total, 95 candidate genes were found in 39 significant SNPs (17 genes were found simultaneously near different SNP sites) (Supplementary Table 9). Among the 95 genes associated with the flowering time, 7 genes were differentially expressed during the bud stage, 13 genes were differentially expressed during the initial flowering stage, and 18 genes were differentially expressed during the full flowering stage. Most significant SNPs were located in intergenic spacer regions. To further identify the candidate genes for flowering time in alfalfa, the DEGs closest to the significantly associated SNPs were analyzed by candidate gene association mapping. According to this criterion, 3 DEGs were included in the subsequent analysis. To verify the accuracy of these results, primers were designed for these three genes for qRT–PCR verification (Supplementary Table 10). The results show that the qRT–PCR results are consistent with the RNA-seq results. The expression levels of these three genes gradually increased in the early flowering cultivars and were relatively decreased in the late-flowering cultivars, indicating that these three genes may be closely related to the growth and development of plants (Figure 6).

**FIGURE 7:**
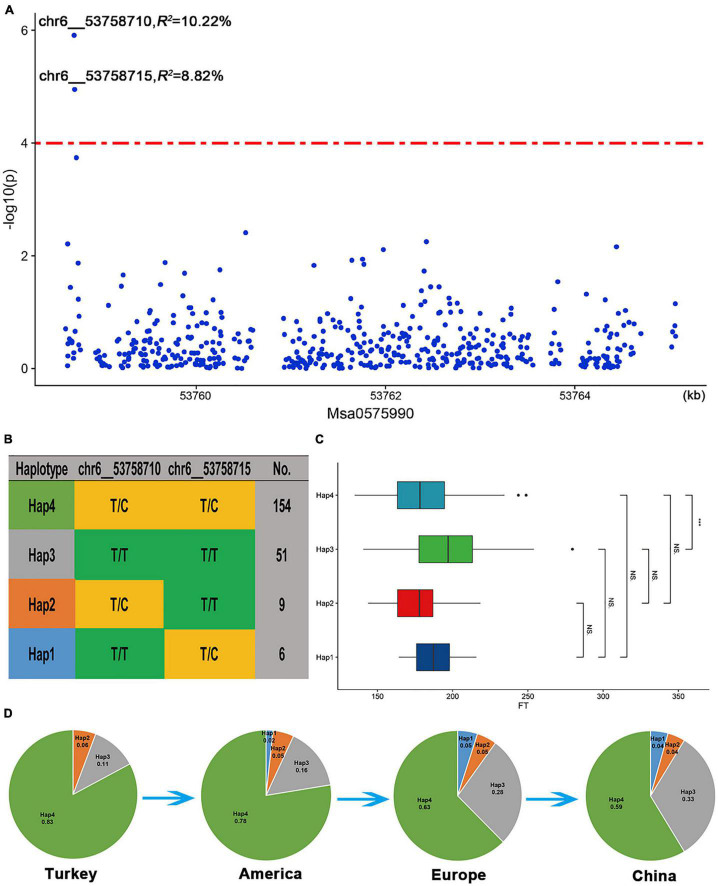
*Msa0888690* involvement in the flowering time in alfalfa. **(A)** Association analysis of SNPs located within the *Msa0888690* gene region. **(B)** Haplotype analysis of significant SNPs associated with the flowering time. **(C)** Comparison of the flowering time among the four haplotypes. A boxplot of the four haplotypes is shown on the left, and the symbols represent the correlations. **(D)** Allele frequency of the four haplotypes in the Turkish, American, European and Chinese subgroups.

Among the three candidate genes, *Msa0888690*, which encodes a protein involving isoprene, was selected for analysis because there were enough SNP sites around this gene. This gene is highly homologous (59.2% identical) to the *A. thaliana AT2G37540* gene, which is involved in the regulation of the development of plant pollen tubes. In addition, we found that two SNPs (chr6__53758710 and chr6__53758715) located approximately 30 kb upstream of the *Msa0888690* gene were significantly related to the flowering time (Figure 7A). The two significant SNPs in the upstream region of *Msa0888690* existed as four different haplotypes in the association panel (Hap1-Hap4) (Figure 7B). Among the four haplotypes, Hap2 (*T/C*, *T/T*) had the lowest frequency; it was the first to bloom, followed by Hap4 (*T/C*, *T/C*) and Hap1 (*T/T*, *T/C*), and the latest was Hap3 (*T/T*, *T/T*) (Figure 7C). Regarding the different geographical origins, the frequency of Hap3 was increased among the American, European and Chinese varieties. In addition, Hap1 was not found among the Turkish varieties, and its frequency gradually increased among the other varieties (Figure 7D). This finding suggests that Hap3 and Hap1 may be under selection in alfalfa breeding.

## Discussion

In plants, the flowering time is an important characteristic that affects yield and quality. The identification of genetic loci for flowering time traits is helpful for clarifying the genetic basis of the growth period, which is of great value for cultivars suitable for different geographical regions. Currently, association analyses and linkage analyses are the two most commonly used methods for locating genes underlying complex traits, and these methods have been widely used in plant breeding. Linkage analyses can be used to eliminate false positives of related loci. Indeed, several studies have investigated the genetic basis of alfalfa flowering (Adhikari et al., 2019; Zhang et al., 2020). However, the intervals between positions are usually large in alfalfa, and association analyses can greatly reduce these intervals. Therefore, accuracy and efficiency can be improved by the combination of these two methods. These studies are important but did not provide enough information to determine the mechanisms underlying the alfalfa flowering time. In this study, we used 875,023 high-quality, high-density SNP markers to analyze the genetic basis of the flowering time via a GWAS of 220 alfalfa materials. We detected numerous significantly associated loci and genes. In addition, through the combination of a transcriptomic analysis and GWAS, several candidate genes related to the flowering time were analyzed. Our results revealed many promising genes for further studies investigating plant growth and development and for breeding new alfalfa varieties suitable for harvesting.

Mastering the proper flowering time can maximize economic benefits (Koornneef et al., 1998a). However, we still do not understand the mechanism underlying the flowering time, and research investigating the influence of the flowering period on alfalfa varieties in different geographical regions and improvement in breeding conditions is limited. The results of the present study showed that there were no significant differences in the flowering time of alfalfa varieties in different geographical regions. However, compared with the varieties from other geographical areas, there are significant differences between Chinese varieties and American varieties. The flowering of Chinese varieties occurs obviously earlier than that of American varieties. The reason for this difference is not only due to climatic conditions but also closely related to the germplasm history. Chinese alfalfa varieties have been cultivated for more than 2000 years and have continuously experienced domestication selection, resulting in several local varieties (Annicchiarico et al., 2015) that are suitable for different ecological environments. In contrast, the genetic diversity of American germplasm materials is low, which may be due to the short introduction time of alfalfa in the United States, which was introduced approximately 200∼300 years ago (Prosperi et al., 2014). The short cultivation history and low diversity of germplasm materials at the time of introduction may lead to great differences in the flowering time between American varieties and Chinese varieties. Our previous research results show that the decay of LD with distance varied as a function of geographical origin: The LD decay distance of American alfalfa varieties is 40.1 kb, and that of Chinese varieties is 17.7 kb (Chen et al., 2021), which further indicates that the breeding improvement status and geographical origin may affect the allelic diversity and Germplasm history of alfalfa.

However, research concerning the flowering time is very scarce, and there is no reference genome for alfalfa; thus, the important markers are aligned only with the reference genome (*M. truncatula*). Therefore, we are concerned that markers associated with important traits may be lost. In the past two years, several alfalfa reference genomes have been released, which has made it possible to mine candidate genes related to flowering traits based on GWAS results (Chen H. et al., 2020; Shen et al., 2020; Long et al., 2022). In this study, we used the average flowering time as an indicator to investigate the flowering period in alfalfa association populations and used three-year data. In total, 67 significant SNP markers were identified in this study. Among these significant SNP makers, we found some SNPs have the higher LOD value but with lower explained variance for example the SNP (chr6__72110460). In general, if a QTL or significant SNP (GWAS) which have a higher LOD value may also have a higher explained variance. The reason for this results in our study maybe that this SNP maybe is false positive and need to be confirmed in the future study. In addition, two SNPs were colocalized in the two environments. Our results revealed a small number of identical SNPs across different phenotypes, which may be due to the large variation in temperature and different day lengths (average day length over three years differed by nearly an hour, approximately 40 min) influencing the alfalfa flowering time during the three years or the absence of major flowering-related alleles in alfalfa. The number of important SNP markers detected by our GWAS indicates that this phenotype can be effectively used to study the flowering period of alfalfa. Moreover, 95 candidate genes were located in 67 markers. Therefore, most markers we found were detected by our GWAS at a single-gene resolution. The annotation analyses of these candidates identified potential mechanisms for the genetic regulation of the alfalfa flowering time. Of these candidate genes, 23% play a role in plant growth and development, and 13% are involved in pollen tube growth (Figure 4). Therefore, these genes may be the main genes that cause flowering time changes in related populations. According to a previous report, plant hormones, such as auxin, cytokinin (CK), abscisic acid (ABA), gibberellic acid (GA), salicylic acid (SA), and jasmonic acid (JA), are involved in the floral transition process and plant flowering (Martínez et al., 2004; Davis, 2009; Bartrina et al., 2011). In our study, 10% of the candidate genes were involved in the response to stress and ABA, suggesting that these processes are also associated with important mechanisms regulating the growth and development of the plants composing this population.

Transcriptomic analyses have been shown to be a powerful tool for identifying genes related to plant growth and development. In recent years, RNA-seq technology has also been used to study alfalfa, and many genes related to important traits have been identified, such as traits associated with defoliation (Cheng et al., 2018), drought stress (Arshad et al., 2018), and salt stress (Postnikova et al., 2013). In the present study, 1,924, 2,405, and 3,779 DEGs were upregulated at different flowering stages, and 1,651, 2,613, and 4,730 DEGs were downregulated. These genes can help us understand the molecular mechanism of alfalfa flowering. In addition, we identified three DEGs in three different developmental stages, all of which play a role in flower growth and development. For example, *Msa1016440* (*AT5G41990*), encoding ATWNK8, regulates the flowering time by regulating the photoperiodic pathway in *A. thaliana* (Wang et al., 2008). ABA is a sesquiterpenoid phytohormone that plays crucial roles in plant development, growth, and responses to stresses (Chen K. et al., 2020; Nonogaki, 2020). Phaseic acid (PA), the main catabolite of ABA, is structurally related to ABA and exhibits ABA-like hormone activity (Nambara and Marion-Poll, 2005). In *A. thaliana* plants overexpressing Cinnamoyl-coA: NADP oxidoreductase-like 1 (CRL1), which encodes a PA reductase that catalyses the formation of dihydrophaseic acid from PA, both the seed germination rate and flowering time were increased (Yin et al., 2022). *Msa0344630* also encodes cinnamoyl-coA, and its expression was significantly increased according to the RNA-seq data. Therefore, we speculate that this gene can also promote the early flowering of plants. This finding shows that these DEGs play an important role in alfalfa flowering.

Isoprenoids constitute the largest class of natural plant products and perform diverse biological functions, including functions in plant growth and development (Lange et al., 2020). Through the overexpression of isoprenoid homologue 3-hydroxy-3-methylglutaryl CoA reductase (HMGR) in *Solanum tuberosum*, it was found that the flowering time was advanced, and biomass was increased. Here, we also found that *Msa0888690*, which encodes a protein involving isoprene, was significantly associated with the flowering time of alfalfa. The expression of *Msa0888690* in alfalfa flowers was significantly upregulated at different flowering stages. Interestingly, we found four haplotypes at this location, with Hap3 flowering the latest and increasing in frequency in different geographical origins, and among the varieties in China, the proportion of Hap3 was the greatest. Hap1 germplasm was not present among the Turkish cultivars but gradually increased in frequency in several other cultivars with different geographical origins. Therefore, we hypothesize that Hap3 and Hap1 may be among the targets selected during the domestication of alfalfa. The generation of transgenic alfalfa with *Msa0888690* knocked out via miR156 or *Msa0888690* overexpressed could facilitate the elucidation of its functions in growth and development. The genetic materials, candidate genes, and haplotypes identified in our study may potentially be used for cultivating alfalfa varieties suitable for local needs.

## Data Availability Statement

The original contributions presented in the study are publicly avlailable. RNA-seq data of the flowers at three development periods of two species of alfalfa grown in the field have been submitted to the NCBI Sequence Read Archive (BioProject: PRJNA817856).

## Author Contributions

JK, RL, and QY planned and designed the research. FH, FZ, and LC analyzed the data and wrote the manuscript. ML, XJ, YC, TG, TY, and CW performed the field work and collected the phenotypic data. ZW, RL, and LC supervised the research. FH and FZ contributed equally. All authors read and approved the final manuscript.

## Conflict of Interest

The authors declare that the research was conducted in the absence of any commercial or financial relationships that could be construed as a potential conflict of interest.

## Publisher’s Note

All claims expressed in this article are solely those of the authors and do not necessarily represent those of their affiliated organizations, or those of the publisher, the editors and the reviewers. Any product that may be evaluated in this article, or claim that may be made by its manufacturer, is not guaranteed or endorsed by the publisher.

## Footnotes

1
http://eggnog5.embl.de/#/app/home

2
https://blast.ncbi.nlm.nih.gov/

## Supplementary Material

Supplementary Figure 1 Normal distribution of the average values of the flowering time over three years among the association panel.

Supplementary Figure 2 Normal distribution of the average values of the flowering time in a single year among the association panel.

Supplementary Figure 3 Correlations between different years. The correlation coefficient (R) and P value are presented at the top of the figure. **(A)** Correlation information in 2019 and 2020. **(B)** Correlation information in 2019 and 2021. **(C)** Correlation information in 2020 and 2021. The gray part represents the 95% confidence interval.

Supplementary Figure 4 GO analysis of all DEGs.

Supplementary Figure 5 GO analysis of the bud stage.

Supplementary Figure 6 GO analysis of the initial flowering stage.

Supplementary Figure 7 GO analysis of the full-flowering stage.

Supplementary Table 1 Significant SNPs identified via BLINK.

Supplementary Table 2 Genes associated with growth and development identified by sequence similarity.

Supplementary Table 3 Upregulated DEGs in the bud stage under normal growth conditions.

Supplementary Table 4 Downregulated DEGs in the bud stage under normal growth conditions.

Supplementary Table 5 Upregulated DEGs in the initial flowering stage under normal growth conditions.

Supplementary Table 6 Downregulated DEGs in the initial flowering stage under normal growth conditions.

Supplementary Table 7 Upregulated DEGs in the full-flowering stage under normal growth conditions.

Supplementary Table 8 Downregulated DEGs in the full-flowering stage under normal growth conditions.

Supplementary Table 9 Candidate genes identified via GWAS and a transcriptomic analysis.

Supplementary Table 10 Primers used in this study.
