# Supplementary figures and images for: A Genome-Wide Association Study Coupled With a Transcriptomic Analysis Reveals the Genetic Loci and Candidate Genes Governing the Flowering Time in Alfalfa (Medicago sativa L.)
Source: Front Plant Sci. 2022 Jul 11;13:913947. doi: 10.3389/fpls.2022.913947 (PMC9310038; doi:10.3389/fpls.2022.913947)

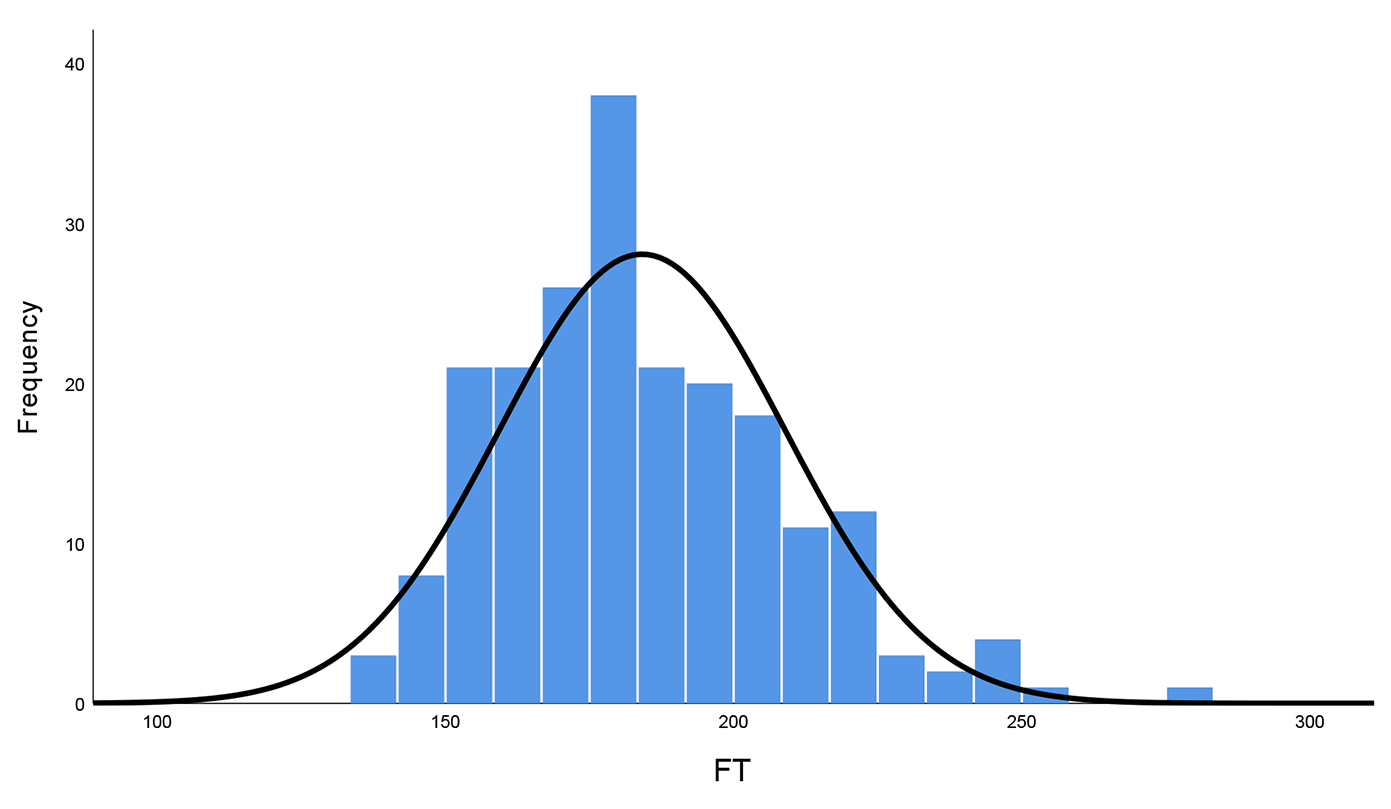

Supplement: Supplementary Figure 1 — Normal distribution of the average values of the flowering time over three years among the association panel. [file Image_1.TIF]

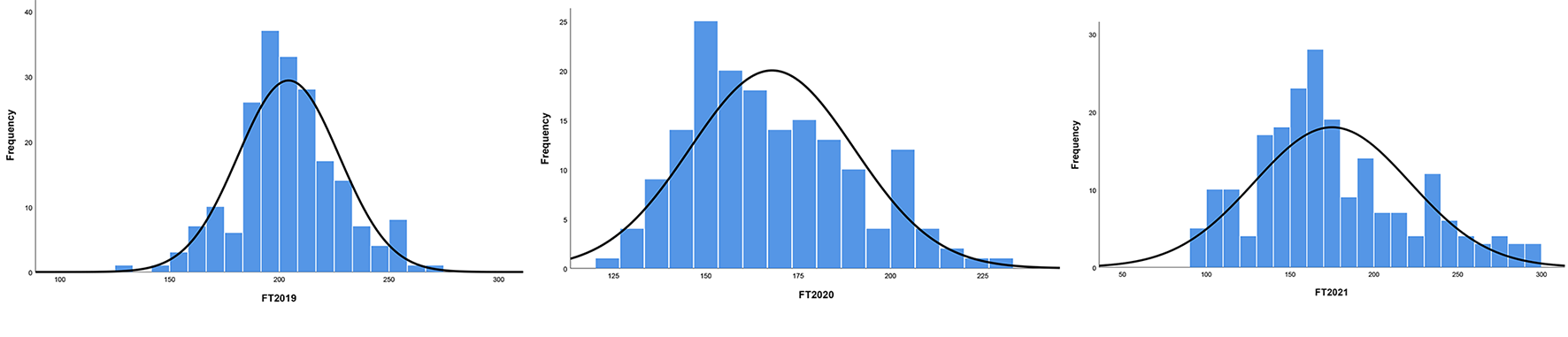

Supplement: Supplementary Figure 2 — Normal distribution of the average values of the flowering time in a single year among the association panel. [file Image_2.TIF]

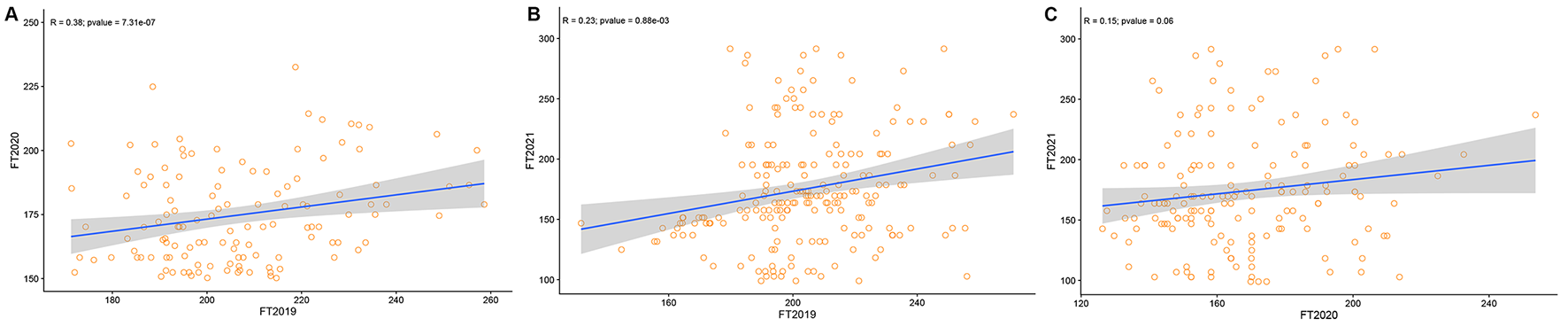

Supplement: Supplementary Figure 3 — Correlations between different years. The correlation coefficient (R) and P value are presented at the top of the figure. (A) Correlation information in 2019 and 2020. (B) Correlation information in 2019 and 2021. (C) Correlation information in 2020 and 2021. The gray part represents the 95% confidence interval. [file Image_3.TIF]

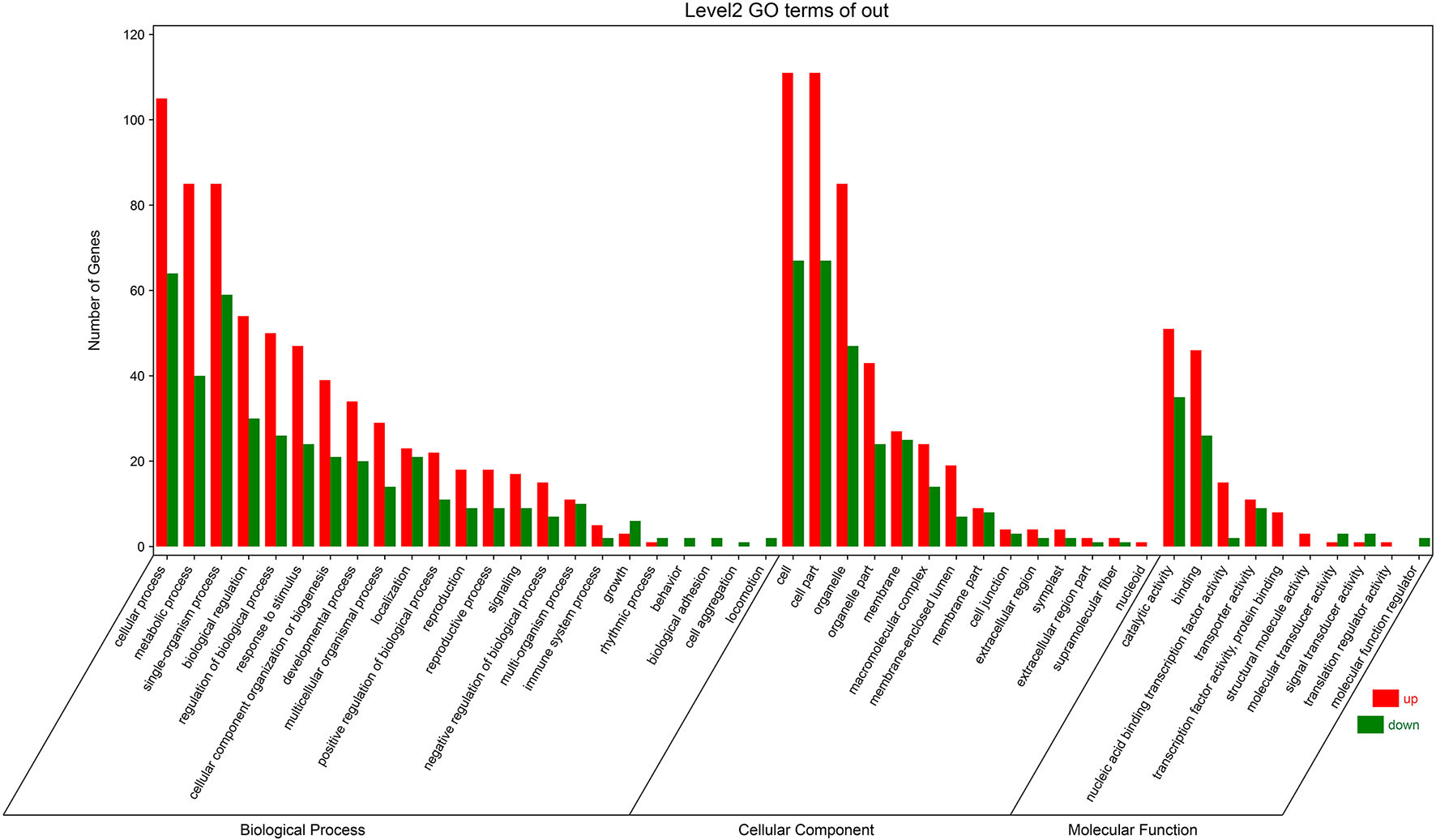

Supplement: Supplementary Figure 4 — GO analysis of all DEGs. [file Image_4.TIF]

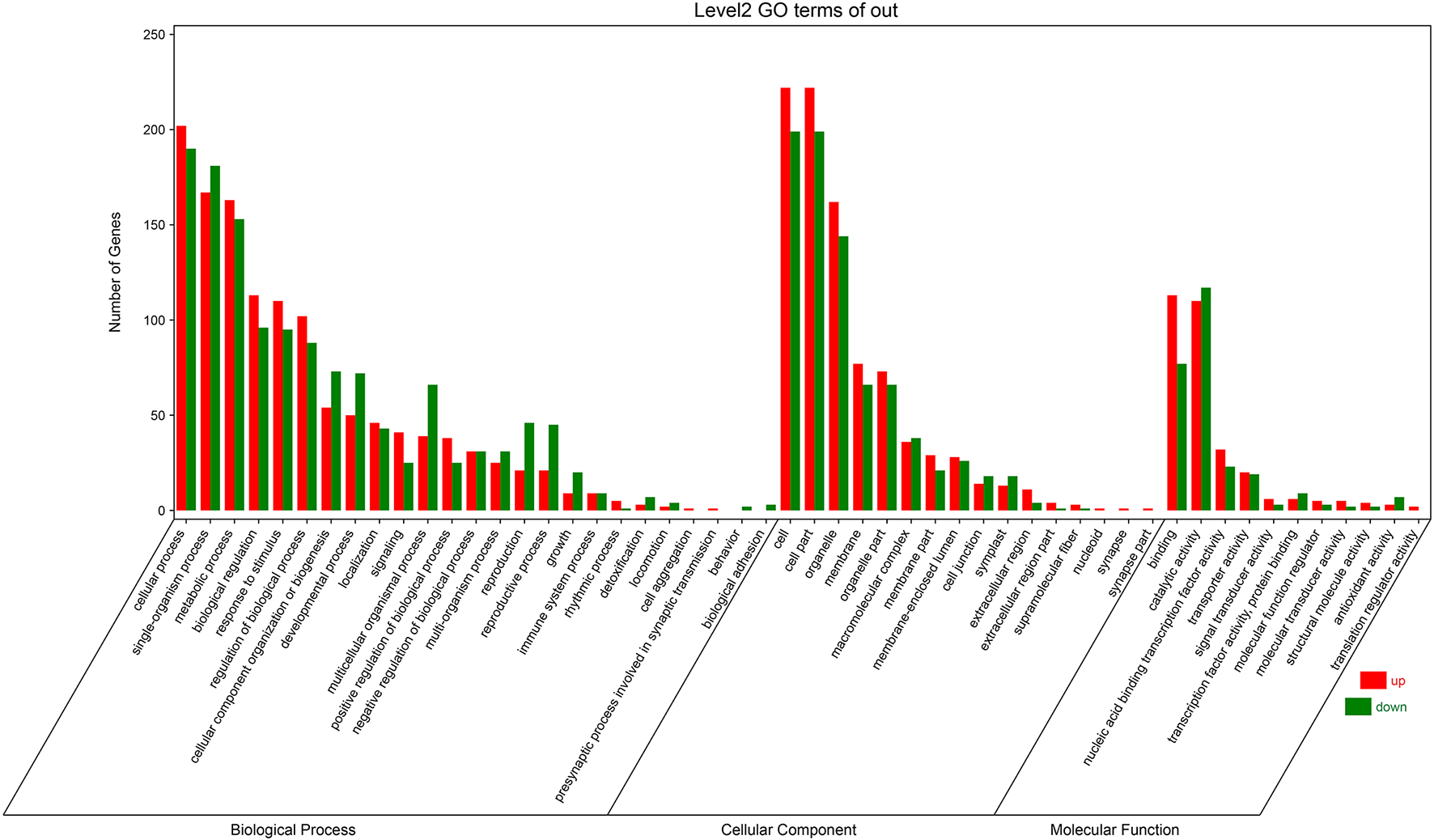

Supplement: Supplementary Figure 5 — GO analysis of the bud stage. [file Image_5.TIF]

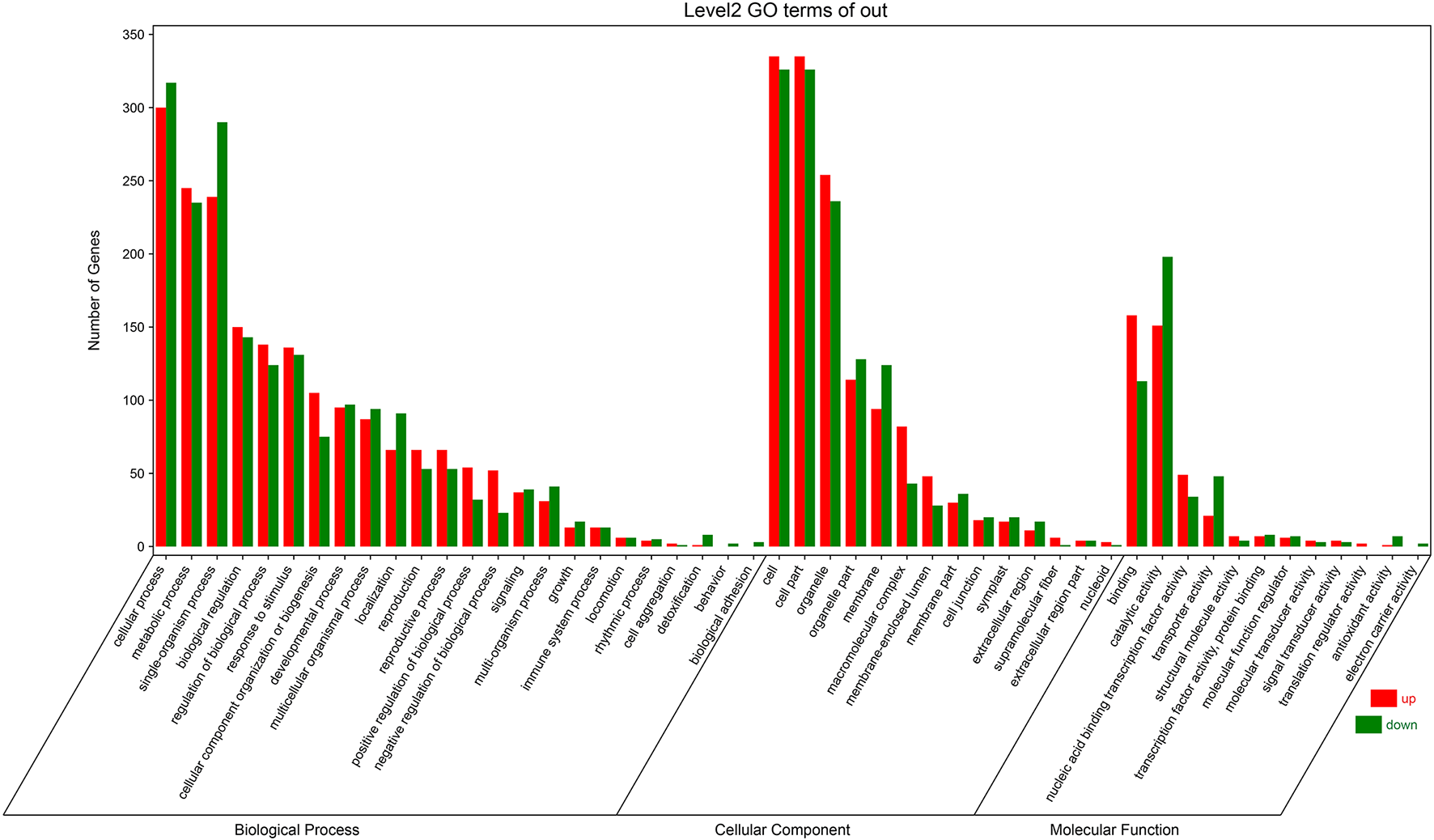

Supplement: Supplementary Figure 6 — GO analysis of the initial flowering stage. [file Image_6.TIF]

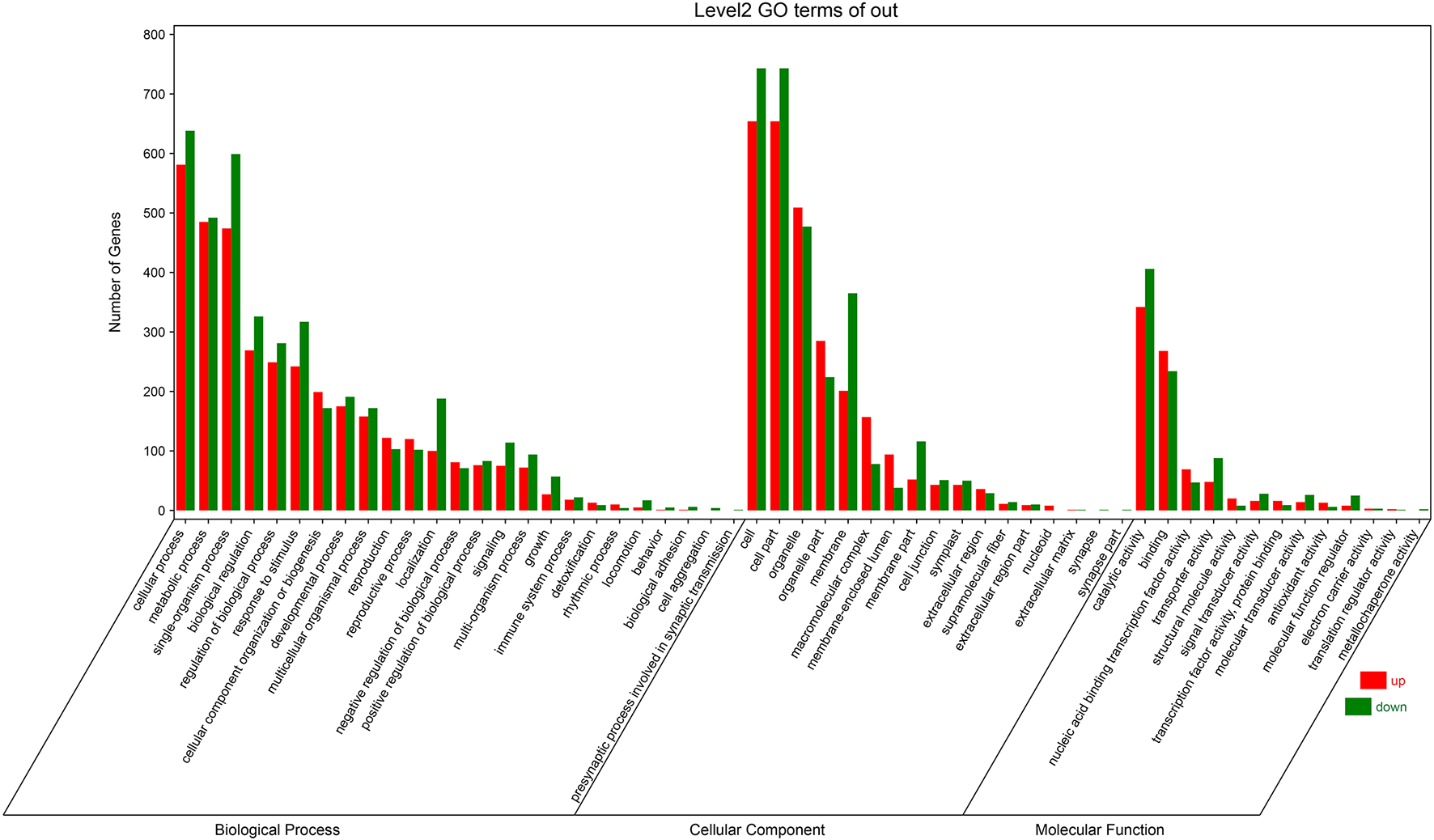

Supplement: Supplementary Figure 7 — GO analysis of the full-flowering stage. [file Image_7.TIF]
